# Information and Childhood Cancer

**Published:** 2016-06-30

**Authors:** Oscar Ramirez Wurttemberger

**Affiliations:** Sistema de Vigilancia Epidemiológica de Cáncer Pediátrico (VIGICANCER), Cali, Colombia; Registro Poblacional de Cáncer de Cali, Cali, Colombia; Fundación POHEMA, Pediatras Oncohematologos, Cali, Colombia

 Currently in Colombia, cancer represents the third leading cause of death in children between 1 to 14 years of age. Although childhood cancer (<15 years old) is a small proportion (0.5-3%) of all cases of cancer in the population, most of these cancers (84%) occur in low and middle-income countries (LMIC), in where 90% of the child population live. The Population-based Cancer Registry of Cali (RPCC) has documented an increase of 0.9% in the annual percentage change in the overall childhood cancer incidence measured from 1977 to 2011 1. 

 Unlike cancer in adults, the control of childhood cancer in the population cannot be based on prevention or in preclinical detection. Therefore, the control of these diseases rests on a rapid and correct diagnosis, and in the effective implementation of treatment. Both diagnoses and treatments are complex, implying a significant challenge for the health system and the society in general. Knowledge about the local clinical outcomes is essential to make informed decisions which would improve chances of cure of these patients. For this, it is essential to have systematic, thorough, valid and timely information to help guide these actions. In this sense, to have systems for the continuous monitoring of clinical outcomes of children with cancer becomes part of the strategy for increase their survival probabilities. Because the implementation of treatments is complex and dependent on local factors, it becomes imperative to have a system to collect data in every city where a pediatric oncology unit exist.

 Recognizing the above, RPCC established in 2009 a monitoring system of clinical outcomes of children with cancer (VIGICANCER), with the support of the program "My Child Matters" of the Sanofi-Espoir Foundation 2. More recently, this system has expanded to Pasto, Neiva, Ibague, Bucaramanga, and it is starting in Cartagena. Since VIGICANCER establishment until December 2015, 1,286 patients have been registered in Cali; and 413 (2013-2015) in other cities. Using this information we estimated that the five-year survival probability of new cases of children with cancer treated in Cali is 52% (95% CI: 48-55). Survival at one, three and five years is 70%, 58%, and 56%, respectively, for children with acute lymphoblastic leukemia (ALL). For cases of acute myeloid leukemia (AML), the survival at one and three years is 45%, and 33% respectively; and then it approaches a plateau. These figures contrast with those published by the Monitoring Group in Public Health from the Ministry of Health, which shows a five-year survival of 85.2% in children with ALL; and a survival of 72.9% at one year for AML 3. 

Why is there this discrepancy? Which of the figures is closer to the Colombian reality and why is this important?

 In 2008, pediatric acute leukemias (PAL), and specifically LLA, became public health priority in Colombia because of the high mortality rates presented within the region. From 2005-2008, the mortality rate (x10^6)^ for PAL in boys was 23, and 20 in girls 4. Unfortunately, these mortality rates remain relatively stable over time (2007-2011), with 22 for boys and 17 for girls 5, About the same period in the U.S.A. 6, (2007-2010), the mortality rate for PAL in this age group was 3; about 6.5 times less than that reported in Colombia. The Hospital-based Children's Cancer Registry of Argentina (ROHA), with a follow-up of 10,181 cases (2000-2007), reported a three-year survival of 69% for ALL 7. Considering the above and according to the estimates published by the Ministry of Health, the survival of children in Colombia with ALL (85%) is higher than in Argentina (69%) and similar (86%) to what is reported in Europe 8. Something similar happens for AML, for which the ROHA describes a three-year survival of 40%. Considering the above data and what is found in VIGICANCER, the most likely explanation is that the figures reported by the Ministry are strongly biased, and therefore, considerably overestimates the survival of these patients. 

 The Ministry report is based on SIVIGILA information. When comparing the cases reported to SIVIGILA (in Cali) with those of VIGICANCER, we found more than 50% of case underreporting in SIVIGILA. This problem of notification is not trivial because both, the treatment center and the type of population served, heavily influences clinical outcomes in cancer. Hence, it cannot be assumed that the reported cases are representative of all the people under treatment. The same report mentions that 162/349 deaths were recorded as deceased before starting the follow-up. Apparently, this group was not included in the survival analysis. The exclusion of these deaths coincides with a median mortality time of 50.9 months. We know that about 30 to 40% of the deaths in children with PAL occur in the first year of treatment. The only possible explanation of having a median mortality time so long is to exclude early deaths. The exclusion of these patients overestimates survival. Moreover, this also means that the followed cases came from a cohort of survivors. This cohort of survivors has less chance of death during follow-up because they are beyond the period of highest mortality risk; overestimating, even more, the survival probabilities. The report says that 415 had no effective contact for lack of data on the SIVIGILA system. This means that 415/1,664 alive children, diagnosed with PAL, could not be followed (24.3%). This implies a very high loss to follow-up. But the most important is the fact is that these losses are not random. Patients who usually do not have information to be contacted are also the ones with the worst results. This has been shown in epidemiological studies in other contexts. We also consider as acceptable to assume that in the group of cases that could not be reached, predominated patients from low socioeconomic status families, without permanent employment, and less social support networks. The factors mentioned above are associated with the abandonment of treatment 9, and therefore with poor survival. Because treatment abandonment is in the causal pathway of death in cancer patients. Finally, the report is intended to be representative of the country's reality but does not take into account that the registration of cases is not representative of all Colombian regions. We believe that these points are the most important to explain the discrepancies between the information published by the Ministry with that of VIGICANCER, the mortality rates, and the international studies. 

 Concerning cancer control by health authorities, it is essential to have reliable and timely data for decision-making. This information is crucial to know the real extent of the problem, to plan strategies for prevention, screening, treatment and rehabilitation. To allocate the necessary resources for investment, and to systematically assess the impact of all the interventions. Therefore, information is an integral part of the efforts to control cancer in populations, including childhood cancer. It is clear that with information disconnected from the Colombian reality, health authorities will not be able to approach the problem in a rational way. 
